# Importance of vaccination against human papillomavirus in a rural settlement in Terenos, Mato Grosso do Sul

**DOI:** 10.11606/s1518-8787.2023057004339

**Published:** 2023-03-15

**Authors:** Zilda Alves de Souza, Marco Antonio Moreira Puga, Inês Aparecida Tozetti, Marcella Naglis de Oliveira Lima, Milena Sonchine de Souza, Marisa de Fátima Lomba de Farias, Estela Márcia Rondina Scandola, Cacilda Tezelli Junqueira Padovani

**Affiliations:** I Universidade Federal de Mato Grosso do Sul Instituto Integrado de Saúde Programa de Pós-graduação em Saúde da Família Campo Grande MS Brasil Universidade Federal de Mato Grosso do Sul. Instituto Integrado de Saúde. Programa de Pós-graduação em Saúde da Família. Campo Grande, MS, Brasil; II Universidade Federal de Mato Grosso do Sul Instituto de Biociências Campo Grande MS Brasil Universidade Federal de Mato Grosso do Sul. Instituto de Biociências. Campo Grande, MS, Brasil; III Universidade Federal de Mato Grosso do Sul Programa de Pós-graduação em Saúde da Família Campo Grande MS Brasil Universidade Federal de Mato Grosso do Sul. Programa de Pós-graduação em Saúde da Família. Campo Grande, MS, Brasil; IV Universidade Federal da Grande Dourados Faculdade de Ciências Humanas Dourados MS Brasil Universidade Federal da Grande Dourados. Faculdade de Ciências Humanas. Dourados, MS, Brasil

**Keywords:** Rural Population, Papillomavirus infections, prevention & control, Vaccination Coverage, Refusal of Vaccination, Knowledge, Attitudes and Practice in Health, Family Health

## Abstract

**OBJECTIVE:**

To understand health professionals’ perceptions about vaccination against human papillomavirus (HPV) in the Santa Mônica rural settlement in Terenos, Mato Grosso do Sul.

**METHODS:**

Quantitative and qualitative methodologies, consultations on vaccination cards, records of community health agents and the focus group technique were used. The main factors of hesitation and vaccine refusal were analyzed, as well as the health team’s strategies for the process of immunization against HPV, from June to August 2018.

**RESULTS:**

Of 121 children and adolescents, 81 (66.94%) received the complete vaccination schedule. Complete vaccination coverage for women was 73.17% (60/82) and for men, 53.8% (21/39). It was observed that, although strategies are adopted for vaccine promotion, such as mobile actions, the public is resistant due to superficial knowledge about the vaccine and its use in an early age group, showing itself to be susceptible to the negative influence of the media and to society’s taboos. In addition, difficulties regarding the use of the Unified Health System card and the shortage of professionals were also observed.

**CONCLUSION:**

The results explain the immunization coverage below the target and reinforce the need to strengthen the family health strategy, as well as the permanent and continuing education of professionals, in order to increase parental confidence and adherence to vaccination.

## INTRODUCTION

Cervical cancer is correlated with human papillomavirus (HPV) infection/, being considered an etiological factor for the development of neoplasms, mainly involving the high-risk oncogenic types HPV16 and HPV18^1^.

A total of 124 countries and territories had already implemented national immunization programs for HPV vaccination by 2019^2^. In Brazil, the Unified Health System included the HPV vaccine in the vaccination schedule through the National Immunization Program (PNI), in 2014. The program includes girls and boys aged 9 to 14 years, patients living with the human immunodeficiency virus (HIV/Aids), transplanted and undergoing chemotherapy and radiotherapy, aged between 9 and 26 years. Recently, it was extended to women up to 45 years old and with immunosuppression^3^.

Since the implementation of the quadrivalent vaccine against HPV in the primary care network, the program has sought to reach the minimum target of 80% vaccine coverage. In this way, it contributes to reducing the incidence and mortality of different types of cancer induced by viral types HPV 16, 18, 6 and 11, including cervical, vulvar, vaginal, penile and anal, and oropharynx cancer, in addition to genital warts^3^.

Children and adolescents residing in settlements must be vaccinated against HPV established by the National Policy for Comprehensive Health of the Rural and Forest Populations, which aims to meet the health care needs of this target population^4^. Brazil has 972,289 families distributed in 9,374 settlements, which corresponds to an area of 87,978,041.18 hectares, destined for agrarian reform settlements. In the State of Mato Grosso do Sul, 27,764 families are distributed in 204 settlements in different municipalities^5^.

In 2014, most Brazilian municipalities reached the target established in the first dose (87%); however, only 32% of them reached the goal in the second dose^6^. Preliminary surveys carried out in the Santa Mônica – Rural II settlement complex, belonging to the municipality of Terenos, in the state of Mato Grosso do Sul (PA Santa Mônica – CUT, FETAGRI and MST), indicated that adherence to vaccination against HPV for the year 2014 did not reach the national target recommended by the Ministry of Health (unpublished data, observed by the authors, 2014).

Given this evidence, this study aims to understand health professionals’ perception about vaccination against HPV in the Santa Mônica settlement complex and contribute to health action measures that can improve this vaccine coverage, analyzing the main hesitation factors or vaccine refusal, as well as the strategies of the local health team for immunization against the human papillomavirus.

## METHODS

This is a descriptive-exploratory study of a quantitative and qualitative nature that took place in the Santa Mônica - Rural II settlement complex, in the municipality of Terenos (MS), from June to August 2018.

The research involved the three existing social forces: Settlement Carlos Ferrari, organized through the single central workers (CUT), with a total of 86 families; Emerson Rodrigues Settlement, constituted by the Landless Rural Workers Movement (MST), with 186 families; Settlement of the Federation of Agricultural Workers (FETAGRI), with a total of 443 families.

In the 715 lots made available for land reform, 599 families are registered and assisted by the family health strategy (ESF), including 1,253 people. Of these, 82 are girls and 39 are boys within the age range for receiving the HPV vaccine. It is worth mentioning that the distance between the river basic health unit (UBSF) and the lots can reach 18 kilometers.

Quantitative data collection for surveying vaccination coverage took place at the health unit itself, through manual records carried out by nine community health agents, after consulting vaccination cards. In this way, it was possible to cover the entire assigned area of the ESF in the Santa Mônica – Rural II complex, divided into nine micro-areas, named numerically from 1 to 9.

The organization of vaccination coverage data (overall and by micro-areas) was carried out using electronic spreadsheets in Microsoft Excel.

All records of girls between March 2014 and March 2018 were considered. For boys, the interval between July 2017 and March 2018 was considered. The target audience was classified, according to their vaccination status, as CV (complete vaccination schedule – two doses); IV (incomplete vaccination schedule – only the first dose) and NV (not vaccinated). Data were also distributed according to sex and age.

Qualitative data on the health professionals’ perception about vaccination against HPV were obtained through a conversation circle at the health unit and records of observations and experiences carried out during the research process.

Of the 18 health unit workers, 12 (two men and ten women) made up the conversation circle, seven community health agents, one nurse, one doctor, one dentist, one dental assistant and one pharmacy attendant. Eight professionals lived in the settlement and four lived elsewhere. For the purposes of recording and demonstrating results, professionals were identified by the letter P followed by a numerical representation (P1 to P12).

The generating questions for the conversation circle were: 1) how is the team organized to achieve vaccination coverage? 2) what difficulties does the team face in achieving the goals? 3) what could facilitate the achievement of goals? 4) How do you perceive the community’s relationship with vaccination? What about the HPV vaccine? and 5) what age group has more and less regularity in HPV vaccination? Why do you believe this happens? Such questions were aimed at exchanging experiences and lessons learned and raising awareness of the problems faced on a daily basis.

The conversation circle, lasting approximately one hour, was recorded. In addition, the participants were asked to authorize and sign the free and informed consent form. After the “formal” closing of the conversation circle, it was noticed that the participants continued to discuss the subject during the collective lunch, when the observations and facts experienced related to the research theme were recorded.

The research was approved by the Research Ethics Committee of the Federal University of Mato Grosso do Sul with approval opinion No. 2,685,410, on May 30, 2018.

## RESULTS AND DISCUSSION

Taking into account that the results expressed in percentages were obtained through the quotient between the number of children of each sex and the total number of children living in the analyzed micro-area(s), it was observed that, of the 121 children and adolescents participating in the research, 81 (66.94%) received the complete vaccination schedule (first and second dose). Complete vaccination coverage for females was 73.17% (60/82), higher than for males, with 53.8% (21/39). These results are higher than those of Mato Grosso do Sul, which reached 51.1% among girls and 46.7% among boys^7^. They are also higher than those found in Brazil, which did not exceed 45.1% among girls aged 9 to 15 years^8^.

The Figure shows that vaccination coverage did not reach 15% in micro-areas 1 and 9 (2/16 and 1/7 individuals, respectively). Micro-areas 4, 6 and 8 did not exceed 65% (7/12, 3/5 and 9/14, respectively). Micro-area 3 approached the recommended target with 73.3% (11/15). Micro-areas 2, 5 and 7, on the other hand, achieved adherence percentages above the recommended, with 85% for micro-area 2 and 100% for micro-areas 5 and 7 (24/28, 11/11 and 13/13, respectively).

**Figure:**
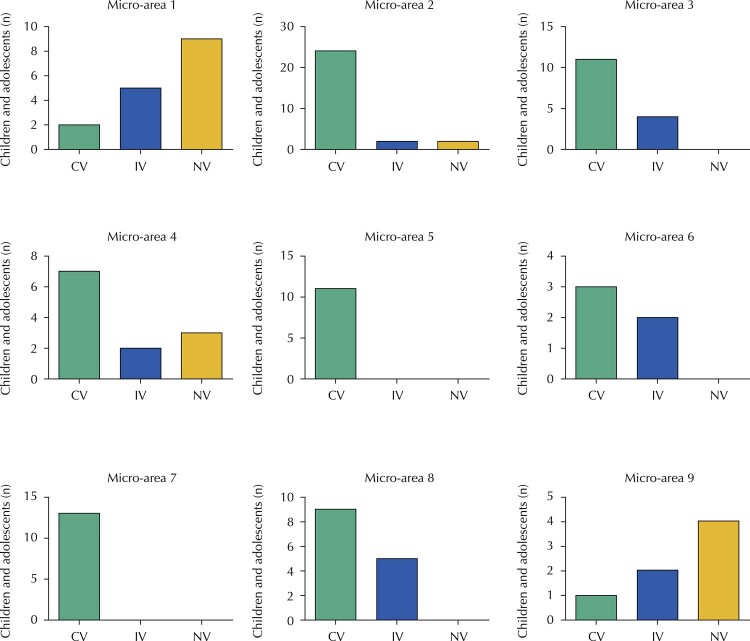
Vaccination coverage by micro-area in the Santa Mônica Settlement, Terenos, 2018. Note: CV: complete vaccination schedule (2 doses); IV: incomplete vaccination schedule (only the 1st dose); NV: unvaccinated.

By observing the distribution of complete vaccination coverage (1st and 2nd doses) of the target population of the study by age according to sex, it is observed that there was no vaccination adherence in the first year for both sexes, gradually increasing with age. However, vaccine adherence decreased in both genders in the last year of vaccine administration ( Table ).

**Table:** Complete vaccination coverage of the female and male public, distributed by age, in the Santa Mônica Settlement, Terenos (MS), 2018 (n = 121).

Public	Age (years)	n	VU	Vaccination coverage (%)
Female	9	11	0	0.00
	10	10	5	50.00
	11	21	19	90.48
	12	12	11	91.67
	13	10	9	90.00
	14	10	10	100.00
	15	8	6	75.00

	Total	82	60	73.17

Male	11	5	0	0.00
	12	5	two	40.00
	13	16	11	68.75
	14	8	6	75.00
	15	5	two	40.00

	Total	39	21	53.85

Source: Data obtained from vaccination cards and health post records.

Note: CV – individuals with complete vaccination schedule (1st and 2nd doses). All records of girls between March 2014 and March 2018 were considered. For boys, the interval between July 2017 and March 2018 was considered.

Several reasons for hesitation or refusal of the vaccine were pointed out by parents and guardians ( Chart ). Vaccination hesitation or refusal in the first year of vaccine administration may be related to the fact that adherence could stimulate sexual activity and contribute to the decrease in condom use and cervical cancer screening. Although many parents believe that vaccination can encourage sexual activity, studies have shown that young women vaccinated against HPV have fewer sexual partners^9^, while other studies have found that vaccination against HPV is not associated with age at first sexual intercourse or the number of sexual partners^10^.

**Chart:** Main reasons for hesitation or refusal of the human papillomavirus vaccine by parents and guardians of children/adolescents pointed out to health professionals, in the Santa Mônica Settlement, Terenos (MS), 2018.

• Lack of information about the vaccine.
• Misinformation transmitted by social networks.
• Fear of serious adverse and side effects after administration of vaccines in general.
• Fear of pain.
• Hesitation or refusal of vaccines in general.
• Lack of trust in health professionals and health agencies.
• Idea that the vaccine can stimulate early sexual activity.
• Idea that vaccines are government strategies to decimate the population.
• Lack of initiative to take the vaccine to the health center, even after educational lectures on the subject.
• Lack of parental initiative, even after other family members reinforced the importance of the vaccine.
• Difficulties using the SUS card (need to be from the municipality of residence, carrying it at the time of vaccination, loss or misplacement).

The fact that the vaccine is exclusively for the primary prevention of a sexually transmitted infection also influences its acceptance^13^. In this study, some parents reported to professionals their difficulties in talking about HPV vaccination with such young girls. However, they reported that the inclusion of boys as a target audience facilitated the approach of children and adolescents about the vaccine. This finding reinforces the need to change paradigms and break family taboos.

In a qualitative analysis, it was observed that less than 10% of knowledge providers knew that HPV vaccination offered some cancer prevention and benefits for men. Thus, the vaccine was rarely offered to boys, which may explain the lower vaccination coverage found in this study^14^.

Mothers’ reports to health professionals in the Santa Mônica Settlement reinforced the importance of reoffering the meningococcal C vaccine in the same age group as the HPV vaccine for boys, as well as expanding the age group for both sexes, in order to expand the number of individuals vaccinated against HPV.

In general, the vaccine adherence of the population in this study was below the recommended target (80%), thus requiring measures to promote adherence to the HPV vaccine. The different realities found across the micro-regions may reflect differences in the involvement of both the population and professionals with vaccine adherence and administration. The low adherence in two micro-areas (1 and 9) can be partly explained by the fact that they are located more than 15 kilometers away from the health unit. The spatial heterogeneity between micro-regions may be one of the reasons for differences in vaccination coverage, which indicates that managers should plan specific strategies for each territory^15^. Women’s lack of knowledge, in rural areas, about how HPV is transmitted and care for prevention can also justify adherence below the target^16^.

The reasons for refusal or hesitation found in this study are consistent with previously reported key issues, such as concerns about the efficacy, safety and possible adverse effects of vaccines, misinformation about related diseases by the target population, parents and health professionals, negative influence of the community, and lack of trust in authorities and pharmaceutical companies^17^.

Since 2012, the World Health Organization has been working to minimize delay in accepting or refusing vaccination despite the availability of vaccine services, through the SAGE Working Group on Vaccine Hesitancy. It is noteworthy that hesitation to vaccinate does not always imply vaccine refusal, since hesitant individuals may accept certain vaccines, but still have doubts about them^18^.

Fear about possible vaccine side effects is also relevant^19^. In this study, it was possible to observe that the negative influence of the media, by circulating erroneous information about the HPV vaccine and its adverse/side effects, led many parents to prohibit their children from being vaccinated.

Another issue that made it difficult was [posts on] Facebook, because they post [people] fainting, feeling sick, catching diseases, then there is the unfortunate coincidence of taking the vaccine and having another type of problem that has nothing to do with it, then they say it is from the vaccine. (P2)

This fact also explains the lower percentage of individuals vaccinated with the complete schedule in micro-areas 1 and 9, as well as the differences in vaccination coverage between the first and second doses^20^.

Beliefs that governments withhold information about side effects have already been reported in some qualitative studies, which negatively influences the campaigns that the Ministry of Health runs to promote the vaccine against HPV^21^.

It is observed that vaccine promotion strategies have undergone changes over the years, after negative consequences generated by the government’s refractory communication in the face of a series of doubts related to the safety or convenience of the vaccine during the first phase of implementation. The inclusion of religious communities, health professionals, families and adolescents as relevant interlocutors, before and during the execution of the first vaccination campaign, could have contributed to better vaccine adherence results^22^.

Currently, access to social media is present in all levels of education and socioeconomic level, including the population of settlements, which may favor the dissemination of information about the importance of the HPV vaccine. On the other hand, the dissemination of false information impairs adherence to vaccination^23^.

Belief in a low risk of contracting HPV or developing cervical cancer correlated with the availability of supposed alternative methods was also pointed out as a reason for the lack of need for the vaccine against HPV, which may partly justify adherence to the vaccine below than recommended^24 , 25^.

Fear of the pain that the vaccine can cause in adolescents was reported by parents and guardians in qualitative studies, as well as fear of the size of the needles and pain during the injection, which is supposed to increase with each dose of the vaccine. Also, the mistaken belief that the vaccine is administered in the cervix, concerns about cleaning the needle and the fear that the injection could lead to loss of virginity were also reported issues^26^.

Although the logistical challenges in health units also create barriers to accessing vaccines in different places, in the present study this difficulty was not observed. Health units had enough doses to serve the entire local target audience and logistical issues do not justify the delay in vaccination or low adherence ^27^.

Another argument reported by parents and professionals for non-adherence to the vaccine was the requirement by the Municipal Health Department that the SUS card from Terenos, municipality of residence, be presented when vaccinating. The main reasons given for the difficulty in acquiring or carrying the SUS card were transportation costs, time and distance to the unit to request the card, and forgetting it on vaccination day. The loss of cards also made it difficult to know the vaccination status of children and adolescents.

Many children and adolescents had the SUS card from the municipality of Campo Grande (MS), which made it impossible to vaccinate in the Santa Mônica settlement, since, for the settled individual to have access to the vaccine, he would have to carry the Terenos card. Although the SUS has universality as a principle when it comes to emergency care, outpatient procedures must be regulated by the municipal reference system; therefore, vaccines must be administered, for the most part, in the municipality of residence^28^.

Community members from the Santa Mônica settlement mentioned many times that they do not transfer the SUS card to the municipality of Terenos, where they legally reside, because practically all specialized services are carried out in Campo Grande, capital of Mato Grosso do Sul, and the most viable route from the settlement to the city of Terenos goes through the capital.

In this study, professionals justified the benefit of mobile actions, pointing out limitations for greater effectiveness of this strategy, which include the need for more vehicles and available professionals.

The mobile action, campaigns in micro-areas, in schools, which get closer to the population. Unfortunately, the Brazilians, they keep postponing it again and again. When you say: “on such day we will be vaccinating, and we’ll be there just for that, you’ll arrive and vaccinate and go away in no time”, then people are more concerned. (P-8)

The exclusive availability of professionals and the agility of the mobile systems encourage adherence to vaccination, unlike care in health units with few professionals, where each of them performs several tasks, resulting in a longer waiting time for care and, consequently, in greater resistance of the family to seek the vaccine.

On the other hand, active search for the target public does not always increase vaccination adherence, as observed in a study carried out in South Africa, where the expected adherence was not achieved even with the introduction of a vaccination program against HPV in schools, in 2014. A possible justification for this result is the parents’ hesitation, a fact capable of causing delays or refusal of the vaccine by the children and adolescents themselves^29^.

Professionals argued that information on changes in the vaccination schedule including priority groups was not passed on even in a newsletter, as is customary and the responsibility of the Municipality of Terenos Health Department.

We only hear a lot about children in this age group, so far nothing has come to us from the Health Department about priority groups. (P4)

There are several cancers with chemotherapy treatment here, and we didn’t pay attention that these people have to take it, I don’t know if this is still valid. (P5)

Because as far as I know, the HPV vaccine is more exclusive for this public of children. (P1)

The competent bodies’ precariousness of communication with this area of difficult access was verified. Professionals’ performance in the settlements’ health unit requires appropriate training that makes use of the understanding of the reality experienced by the rural population, covering the local specificity. It is recommended to prioritize the performance of professionals existing in the community, as they bring with them the experiences and paths taken and, therefore, are able to understand the working dynamics of women and men in/of the field, as well as the distance from this territorialization to the access to that service.

In the present study, health workers carried out relevant reflections and created perspectives for new attitudes in the health context, such as vaccination, carefulness with users, the problems they experienced as well because they were themselves, for the most part, settlement residents. The conversation circle also allowed elements to be listed in health promotion, such as new rules for the vaccine against HPV, which were added to the knowledge already acquired. Thus, it can be stated that the conversation circle cannot be considered only a form of data collection, but, above all, an educational process with equalization of knowledge and decision making by the participants.

The results found reveal the need for a careful analysis of the factors that may have influenced vaccine adherence below the recommended level, with a view to restructuring the strategy of the national vaccination policy for the target population. It has already been shown that intervention practices to increase health professionals’ knowledge about the epidemiology of HPV and its relationship with cancer are essential to increase the rate of recommendation and adherence to the vaccine, and may improve behavior with other immunizations^14^.

Joint action between the education, health and population sectors is necessary for the population to become aware of the importance of immunization against HPV^27 , 30^. The training of professionals should encourage their commitment to the objective of guiding public policies aimed at health care for the agrarian reform population. The reality observed in this study can serve as a basis for a better understanding of the reality of other existing settlements in Brazil.

These policies need to clarify doubts that can lead to distrust and anticipation factors that cause hesitation or vaccine refusal. Equally important, local research is conducted to better understand HPV vaccination hesitancy and other determinants of adoption to inform and shape national policy.

Studies that specifically explore educational interventions for health professionals are still limited, but it is known that nurses and family physicians are commonly referred to in qualitative studies as those who most influence decisions about vaccination against HPV. In addition, the influence of family, parents of other children and friends who have not been vaccinated or who recommended the vaccine is also observed^30^.

The results found in this population reinforce the need for actions that promote knowledge about HPV and vaccination. The strengthening of the ESF and the permanent and continuous education of professionals, as well as the preparation and distribution of explanatory material to the community, will certainly contribute to increase parents’ confidence in relation to their children’s vaccination.
