# Supplementary material for: Importance of vaccination against human papillomavirus in a rural settlement in Terenos, Mato Grosso do Sul
Source: Rev Saude Publica. 2023 Mar 15;57:10. doi: 10.11606/s1518-8787.2023057004339 (PMC10118412; doi:10.11606/s1518-8787.2023057004339)
Supplement: Translation

# Translation: Importância da vacinação contra o papilomavírus humano em um assentamento rural em Terenos, Mato Grosso do Sul

**Authors:** Zilda Alves de Souza, Marco Antonio Moreira Puga, Inês Aparecida Tozetti, Marcella Naglis de Oliveira Lima, Milena Sonchine de Souza, Marisa de Fátima Lomba de Farias, Estela Márcia Rondina Scandola, Cacilda Tezelli Junqueira Padovani

**Affiliations:** I Brasil Universidade Federal de Mato Grosso do Sul. Instituto Integrado de Saúde. Programa de Pós-graduação em Saúde da Família. Campo Grande, MS, Brasil; II Brasil Universidade Federal de Mato Grosso do Sul. Instituto de Biociências. Campo Grande, MS, Brasil; III Brasil Universidade Federal de Mato Grosso do Sul. Programa de Pós-graduação em Saúde da Família. Campo Grande, MS, Brasil; IV Brasil Universidade Federal da Grande Dourados. Faculdade de Ciências Humanas. Dourados, MS, Brasil

**Keywords:** População Rural, Infecções por Papillomavirus, prevenção & controle, Cobertura Vacinal, Recusa de Vacinação, Conhecimentos, Atitudes, Prática em Saúde, Saúde da família

## Abstract

**OBJETIVO:**

Compreender as percepções dos profissionais de saúde acerca da vacinação contra o papilomavírus humano (HPV) no Complexo de assentamentos Santa Mônica, em Terenos, Mato Grosso do Sul.

**MÉTODOS:**

Foram utilizadas metodologias quanti-qualitativas, consultas em cartões vacinais, registros de agente comunitários de saúde e a técnica de grupo focal. Foram analisados os principais fatores de hesitação e recusa vacinal, bem como as estratégias da equipe de saúde para o processo de imunização contra o HPV, de junho a agosto de 2018.

**RESULTADOS:**

De 121 crianças e adolescentes, 81 (66,94%) receberam o esquema vacinal completo. A cobertura vacinal completa feminina foi de 73,17% (60/82) e a masculina de 53,8% (21/39). Observou-se que, embora sejam adotadas estratégias para a promoção da vacina, como ações volantes, o público encontra-se resistente devido ao conhecimento superficial sobre a vacina e sua utilização em faixa etária precoce, mostrando-se suscetível à influência negativa da mídia e aos tabus da sociedade. Além disso, dificuldades quanto ao uso do cartão do Sistema Único de Saúde e a escassez de profissionais também foram observadas.

**CONCLUSÃO:**

Os resultados justificam a cobertura vacinal abaixo da meta e reforçam a necessidade de fortalecimento da estratégia de saúde da família, bem como da educação permanente e continuada dos profissionais, a fim de aumentar a confiança dos pais e a adesão à vacinação.

## INTRODUÇÃO

O câncer cervical é correlacionado com a infecção pelo papilomavírus humano (HPV), sendo considerado um fator etiológico para o desenvolvimento de neoplasias, com envolvimento, principalmente, dos tipos de alto risco oncogênico HPV16 e HPV18^1^.

Um total de 124 países e territórios já havia implementado programas nacionais de imunização para a vacinação contra o HPV até 2019^2^. No Brasil, o Sistema Único de Saúde incluiu a vacina contra o HPV no calendário vacinal por intermédio do Programa Nacional de Imunização (PNI), no ano de 2014. O programa contempla meninas e meninos de 9 a 14 anos, pacientes vivendo com o vírus da imunodeficiência humana (HIV/Aids), transplantados e em quimioterapia e radioterapia com faixa etária de 9 a 26 anos. Recentemente, houve a ampliação para mulheres de até 45 anos e com imunossupressão^3^.

Desde a implantação da vacina quadrivalente contra o HPV na rede de atenção primária, o programa tem buscado alcançar a meta mínima de 80% de cobertura vacinal. Dessa forma, contribui para a redução da incidência e mortalidade por diferentes tipos de cânceres induzidos pelos tipos virais HPV 16, 18, 6 e 11, dentre eles o de colo do útero, o vulvar, o vaginal, no pênis e região anal, de orofaringe, além das verrugas genitais^3^.

Crianças e adolescentes residentes em assentamentos devem ter assegurada a vacinação contra o HPV estabelecida pela Política Nacional de Saúde Integral das Populações do Campo e da Floresta, que visa atender as necessidades de atenção à saúde desse público-alvo^4^. O Brasil possui 972.289 famílias distribuídas em 9.374 assentamentos, o que corresponde a uma área de 87.978.041,18 hectares, destinadas para assentamentos de reforma agrária. No Estado do Mato Grosso do Sul, 27.764 famílias estão distribuídas em 204 assentamentos em diversos municípios^5^.

Em 2014, grande parte dos municípios brasileiros atingiu a meta preconizada na primeira dose (87%); entretanto, apenas 32% deles atingiram a meta na segunda dose^6^. Levantamentos preliminares realizados no complexo de assentamentos Santa Mônica – Rural II, pertencente ao município de Terenos, no estado de Mato Grosso do Sul (PA Santa Mônica – CUT, FETAGRI e MST), indicaram que a adesão à vacinação contra o HPV para o ano de 2014 não alcançou a meta nacional preconizada pelo Ministério da Saúde (dados não publicados, observados pelos autores, 2014).

Diante dessas evidências, este trabalho tem o objetivo de compreender a percepção dos profissionais de saúde acerca da vacinação contra o HPV no complexo de assentamentos Santa Mônica e contribuir com medidas de ação em saúde que possam melhorar essa cobertura vacinal, analisando os principais fatores de hesitação ou recusa vacinal, bem como as estratégias da equipe de saúde local para imunização contra o papilomavírus humano.

## MÉTODOS

Trata-se de um estudo descritivo-exploratório de natureza quantitativa e qualitativa ocorrido no complexo de assentamentos Santa Mônica – Rural II, no município de Terenos (MS), no período de junho a agosto de 2018.

A pesquisa envolveu as três forças sociais existentes: Assentamento Carlos Ferrari, organizado por meio da central única dos trabalhadores (CUT), com um total de 86 famílias; Assentamento Emerson Rodrigues, constituído pelo movimento dos trabalhadores rurais sem terra (MST), com 186 famílias; Assentamento da Federação dos Trabalhadores da Agricultura (FETAGRI), com um total de 443 famílias.

Nos 715 lotes disponibilizados para reforma agrária, 599 famílias estão cadastradas e assistidas pela estratégia de saúde da família (ESF), incluindo 1.253 pessoas. Destas, 82 são meninas e 39 são meninos dentro da faixa etária de contemplação da vacina contra o HPV. Vale ressaltar que a distância entre a unidade básica de saúde fluvial (UBSF) e os lotes pode chegar a 18 quilômetros.

A coleta de dados quantitativos para levantamento da cobertura vacinal ocorreu na própria unidade de saúde, por meio dos registros manuais realizados por nove agentes comunitários de saúde, após consulta em cartões vacinais. Dessa forma, foi possível cobrir toda a área adstrita da ESF no complexo Santa Mônica – Rural II, dividida em nove microáreas, nomeadas numericamente de 1 a 9.

A organização dos dados de cobertura vacinal (geral e por microáreas) se deu por meio da confecção de planilhas eletrônicas em Microsoft Excel.

Foram considerados todos os registros de meninas entre março de 2014 e março de 2018. Para os meninos foi considerado o intervalo entre julho de 2017 a março de 2018. O público-alvo foi classificado, conforme seu *status* vacinal, como VC (esquema vacinal completo – duas doses); VI (esquema vacinal incompleto – somente a primeira dose) e NV (não vacinados). Os dados também foram distribuídos segundo o sexo e a idade.

Os dados qualitativos sobre a percepção dos profissionais de saúde acerca da vacinação contra o HPV foram obtidos por meio de uma roda de conversa na unidade de saúde e de registros de observações e vivências realizadas durante o processo de pesquisa.

Dos 18 trabalhadores da unidade de saúde, 12 (dois homens e dez mulheres) compuseram a roda de conversa, sendo sete agentes comunitários de saúde, um enfermeiro, uma médica, um odontólogo, um auxiliar de odontologia e um atendente de farmácia. Oito profissionais eram moradores no assentamento e quatro moravam em outros locais. Para fins de registro e demonstração dos resultados, os profissionais foram identificados pela letra P seguida de uma representação numérica (P1 a P12).

As questões geradoras para a roda de conversa foram: 1) como a equipe se organiza para atingir a cobertura vacinal? 2) que dificuldades a equipe enfrenta para atingir as metas? 3) o que poderia facilitar o atingimento das metas? 4) como percebem a relação da comunidade com a vacinação? E com a vacina do HPV? e 5) que faixa etária tem maior e menor regularidade na vacinação do HPV? Por que acreditam que isso ocorra? Tais questões tiveram como objetivo a troca de experiências e aprendizados e a conscientização dos problemas enfrentados cotidianamente.

A roda de conversa, com duração de aproximadamente uma hora, foi gravada. Além disso, pediu-se a autorização e a assinatura do termo de consentimento livre e esclarecido aos participantes. Após o encerramento “formal” da roda de conversa, percebeu-se que os participantes continuaram debatendo o assunto durante a merenda coletiva, momento em que foram realizados os registros das observações e fatos vivenciados relacionados ao tema da pesquisa.

A pesquisa teve aprovação do Comitê de Ética em Pesquisa da Universidade Federal de Mato Grosso do Sul com parecer de aprovação nº 2.685.410, em 30 de maio de 2018.

## RESULTADOS E DISCUSSÃO

Levando em conta que os resultados expressados em porcentagens foram obtidos por meio do quociente entre o número de crianças de cada sexo e o número total de crianças moradoras da(s) microárea(s) analisada(s), foi observado que, das 121 crianças e adolescentes participantes da pesquisa, 81 (66,94%) receberam o esquema vacinal completo (primeira e segunda dose). A cobertura vacinal completa feminina foi de 73,17% (60/82), superior à masculina, com 53,8% (21/39). Esses resultados são superiores aos de Mato Grosso do Sul, que atingiram 51,1% entre meninas e 46,7% entre meninos^7^. Também são superiores aos encontrados no Brasil, que não ultrapassaram 45,1% entre meninas de 9 a 15 anos^8^.

A Figura mostra que a cobertura vacinal não atingiu 15% nas microáreas 1 e 9 (2/16 e 1/7 indivíduos, respectivamente). As microáreas 4, 6 e 8 não ultrapassaram 65% (7/12, 3/5 e 9/14, respectivamente). A microárea 3 se aproximou da meta preconizada com 73,3% (11/15). Já as microáreas 2, 5 e 7 atingiram percentuais de adesão acima do preconizado, com 85% para a microárea 2 e 100% para as microáreas 5 e 7 (24/28, 11/11 e 13/13, respectivamente).

**Figura:** Cobertura vacinal por microárea no Assentamento Santa Mônica, Terenos, 2018. Nota: VC: esquema vacinal completo (2 doses); VI: esquema vacinal incompleto (somente a 1ª dose); NV: não vacinados.

Ao observar a distribuição da cobertura vacinal completa (1ª e 2ª doses) da população-alvo do estudo por idade segundo o sexo, observa-se que não houve adesão vacinal no primeiro ano para ambos os sexos, aumentando gradativamente conforme a idade. No entanto, a adesão vacinal decresceu em ambos os sexos no último ano de contemplação da vacina ( Tabela ).

**Tabela:** Cobertura vacinal completa do público feminino e masculino distribuída por idade, no Assentamento Santa Mônica, Terenos (MS), 2018 (n = 121).

Público	Idade (anos)	n	VC	Cobertura vacinal (%)
Feminino	9	11	0	0,00
	10	10	5	50,00
	11	21	19	90,48
	12	12	11	91,67
	13	10	9	90,00
	14	10	10	100,00
	15	8	6	75,00

	**Total**	**82**	**60**	**73,17**

Masculino	11	5	0	0,00
	12	5	2	40,00
	13	16	11	68,75
	14	8	6	75,00
	15	5	2	40,00

	**Total**	**39**	**21**	**53,85**

Fonte: Dados obtidos a partir de cartões vacinais e registros do posto de saúde.

Nota: VC – indivíduos com esquema vacinal completo (1º e 2º doses). Foram considerados todos os registros de meninas entre março de 2014 e março de 2018. Para os meninos foi considerado o intervalo entre julho de 2017 e março de 2018.

Diversos motivos para hesitação ou recusa à vacina foram apontados por pais e responsáveis ( Quadro ). A hesitação ou recusa vacinal no primeiro ano de contemplação da vacina pode estar relacionada ao fato de que a adesão poderia estimular a atividade sexual e contribuir para a diminuição do uso de preservativos e do rastreamento do câncer do colo do útero. Embora muitos pais acreditem que a vacinação possa estimular a atividade sexual, estudos demonstram que mulheres jovens vacinadas contra o HPV têm menos parceiros sexuais^9^, enquanto outros estudos descobriram que a vacinação contra o HPV não está associada à idade da primeira relação sexual ou ao número de parceiros sexuais^10^.

**Quadro:** Principais motivos de hesitação ou recusa à vacina contra o papilomavírus humano por pais e responsáveis de crianças/adolescentes apontados aos profissionais de saúde, no Assentamento Santa Mônica, Terenos (MS), 2018.

• Falta de informação sobre a vacina.
• Informações equivocadas transmitidas por redes sociais.
• Medo dos efeitos adversos e colaterais graves após administração das vacinas em geral.
• Medo da dor.
• Hesitação ou recusa às vacinas em geral.
• Falta de confiança nos profissionais de saúde e nos órgãos de saúde.
• Ideia de que a vacina pode estimular a atividade sexual precoce.
• Ideia de que as vacinas são estratégias do governo para dizimar a população.
• Falta de iniciativa para levar ao posto de saúde para administração da vacina, mesmo após palestras educativas sobre o tema.
• Falta de iniciativa dos pais, mesmo após outros familiares reforçarem a importância da vacina.
• Dificuldades com o uso do cartão do SUS (necessidade de ser do município de residência, porte no momento da vacinação, perda ou extravios).

O fato de a vacina ser exclusiva para prevenção primária de uma infecção sexualmente transmissível também influencia na sua aceitação^13^. Neste estudo, alguns pais relataram aos profissionais suas dificuldades em falar sobre a vacinação contra o HPV com meninas tão jovens. Entretanto, relataram que a inclusão dos meninos como público-alvo facilitou a abordagem das crianças e adolescentes acerca da vacina. Tal constatação reforça a necessidade de mudança de paradigmas e rompimento de tabus familiares.

Em uma análise qualitativa, foi observado que menos de 10% dos provedores de conhecimento sabiam que a vacinação contra o HPV oferecia alguma prevenção ao câncer e benefícios para os homens. Dessa forma, a vacina foi pouco ofertada aos meninos, o que pode justificar as menores coberturas vacinais encontradas neste estudo^14^.

Relatos de mães aos profissionais de saúde do Assentamento Santa Mônica reforçaram a importância da reoferta da vacina meningocócica C na mesma faixa etária da vacina contra o HPV para meninos, assim como a ampliação da faixa etária para ambos os sexos, a fim de expandir o número de indivíduos vacinados contra o HPV.

Em geral, a adesão vacinal da população deste estudo demonstrou-se abaixo da meta preconizada (80%), necessitando, portanto, de medidas que promovam a adesão à vacina contra o HPV. As diferentes realidades encontradas entre as microrregiões podem refletir diferenças no envolvimento tanto da população quanto dos profissionais com a adesão e administração da vacina. A baixa adesão em duas microáreas (1 e 9) pode ser justificada, em parte, por se localizarem a mais de 15 quilômetros de distância da unidade de saúde. A heterogeneidade espacial entre microrregiões pode ser uma das razões para as diferenças na cobertura vacinal, o que indica que gestores devem planejar estratégias específicas para cada território^15^. O déficit de conhecimento das mulheres, em zonas rurais, sobre a forma de transmissão do HPV e cuidados para a prevenção também podem justificar a adesão abaixo da meta^16^.

Os motivos de recusa ou hesitação encontrados neste estudo são concordantes com questões-chave relatadas anteriormente, como preocupações quanto à eficácia, segurança e possíveis efeitos adversos das vacinas, desinformação sobre as doenças relacionadas por parte da população alvo, pais e profissionais de saúde, influência negativa da comunidade e falta de confiança nas autoridades e empresas farmacêuticas^17^.

Desde 2012, a Organização Mundial de Saúde tem trabalhado para minimizar o atraso na aceitação ou recusa da vacinação a despeito da disponibilidade de serviços de vacinas, por meio do grupo *SAGE Working Group on Vaccine Hesitancy* . Ressalta-se que a hesitação em vacinar nem sempre implica na recusa da vacina, uma vez que indivíduos hesitantes podem aceitar certas vacinas, mas ainda têm dúvidas sobre elas^18^.

O medo sobre possíveis efeitos colaterais da vacina também se mostra relevante^19^. Neste estudo foi possível observar que a influência negativa da mídia, ao circular informações equivocadas sobre a vacina contra o HPV e seus efeitos adversos/colaterais, fez com que muitos pais proibissem seus filhos de serem vacinados.

Outra questão que dificultou foi o tal do *facebook* , porque eles postam desmaiando, passando mal, pegam doenças, daí dá a infeliz coincidência de tomar a vacina e dá um outro tipo de problema que não tem nada a ver, daí eles falam que é da vacina. (P2)

Esse fato também justifica o menor percentual de indivíduos vacinados com esquema completo nas microáreas 1 e 9, bem como as diferenças de cobertura vacinal entre a primeira e a segunda dose^20^.

As crenças de que os governos retêm informações sobre efeitos colaterais já foram relatadas em alguns estudos qualitativos, o que influencia negativamente nas campanhas que o Ministério da Saúde faz para promover a vacina contra o HPV^21^.

Observa-se que as estratégias de promoção à vacina têm sofrido modificações ao longo dos anos, após consequências negativas geradas pela comunicação refratária do governo frente a uma série de dúvidas relacionadas à segurança ou conveniência da vacina durante a primeira fase de implantação. A inclusão das comunidades religiosas, profissionais de saúde, famílias e adolescentes como interlocutores relevantes, antes e durante a execução da primeira campanha de vacinação, poderia ter contribuído para melhores resultados de adesão vacinal^22^.

Atualmente, o acesso às mídias sociais se faz presente em todas as faixas de escolaridade e nível socioeconômico, inclusive na população dos assentamentos, o que pode favorecer a disseminação de informações sobre a importância da vacina contra o HPV. Em contrapartida, a veiculação de informações falsas prejudica a adesão à vacinação^23^.

A crença em um baixo risco de contrair HPV ou desenvolver o câncer cervical correlacionado com a disponibilidade de supostos métodos alternativos também foi apontada como motivo para falta de necessidade da vacina contra o HPV, o que pode justificar, em parte, a adesão à vacina abaixo do preconizado^24 , 25^.

O medo da dor que a vacina pode causar nos adolescentes foi relatado por pais e responsáveis em estudos qualitativos, assim como o medo do tamanho das agulhas e a dor durante a injeção, que supostamente aumenta a cada dose da vacina. Ainda, a crença equivocada que a vacina seja administrada no colo do útero, preocupações com a limpeza da agulha e o medo de que a injeção possa levar à perda da virgindade também foram questões relatadas^26^.

Embora os desafios logísticos em unidades de saúde também criem barreiras no acesso às vacinas em diversos locais, no presente estudo essa dificuldade não foi observada. As unidades de saúde dispunham de doses suficientes para atender a todo o público-alvo local e as questões logísticas não justificam o atraso na vacinação ou baixa adesão^27^.

Outro argumento relatado pelos pais e profissionais para a não adesão à vacina foi a exigência da Secretaria Municipal de Saúde de que se apresentasse o cartão do SUS de Terenos, município de residência, no ato de vacinar. Os principais motivos apontados para a dificuldade em adquirir ou portar o cartão do SUS foram os custos de transporte, o tempo e a distância até a unidade para solicitar o cartão e, ainda, o seu esquecimento no dia da vacinação. O extravio dos cartões também dificultou o conhecimento do *status* vacinal das crianças e adolescentes.

Muitas crianças e adolescentes possuíam o cartão SUS do município de Campo Grande (MS) o que impossibilitava a vacinação no assentamento Santa Mônica, já que, para o indivíduo assentado ter acesso à vacina, deveria portar o cartão de Terenos. Embora o SUS tenha como princípio a universalidade quando se trata do atendimento de urgência, os procedimentos ambulatoriais devem ser regulados pelo sistema municipal de referência; por isso, as vacinas devem ser administradas, em sua grande maioria, no município de residência^28^.

Integrantes da comunidade do assentamento Santa Mônica mencionaram muitas vezes que não transferem o cartão SUS para o município de Terenos, onde juridicamente residem, pelo fato de que praticamente todos os serviços especializados são realizados em Campo Grande, capital de Mato Grosso do Sul, e que o trajeto mais viável do assentamento até a cidade de Terenos passa pela capital.

Neste estudo, os profissionais justificaram o benefício das ações volantes, apontando limitações para a maior eficácia dessa estratégia que incluem a necessidade de mais veículos e profissionais disponíveis.

O volante, as campanhas nas microáreas, nas escolas, que chegam mais perto da população. Infelizmente o brasileiro, vai deixando, vai deixando. Quando você fala: “tal dia vai estar vacinando, e a gente vai estar só pra isso, você vai chegar e vacinar ‘rapidão’ e já vai embora”, daí as pessoas se preocupam mais. (P-8)

A disponibilidade exclusiva dos profissionais e a agilidade dos sistemas volantes estimulam a adesão à vacinação, ao contrário do atendimento nas unidades de saúde com poucos profissionais, em que cada um deles exerce várias atribuições, resultando em maior tempo de espera no atendimento e, consequentemente, em maior resistência da família a procurar a vacina.

Por outro lado, a busca ativa do público-alvo nem sempre aumenta a adesão à vacinação, como observado em estudo realizado na África do Sul, onde não foi atingida a adesão esperada, mesmo com a introdução de um programa de vacinação contra o HPV nas escolas, em 2014. Uma possível justificativa para esse resultado é a hesitação dos pais, fato capaz de gerar atrasos ou recusa à vacina por parte das próprias crianças e adolescentes^29^.

Os profissionais argumentaram que as informações das mudanças no calendário vacinal com inclusão de grupos prioritários não foram repassadas nem mesmo em boletim, como é de costume e de responsabilidade da Secretaria de Saúde do Município de Terenos.

A gente só ouve falar muito das crianças na faixa etária, até então não chegou nada da Secretaria de Saúde pra nós sobre grupos prioritários. (P4)

Aqui tem vários cânceres com tratamento quimioterápico, e a gente não se atentou que estas pessoas têm que tomar, não sei se isso ainda está valendo. (P5)

Porque até onde eu sei a vacina do HPV é mais exclusiva pra este público de crianças. (P1)

Foi constatada a precariedade da comunicação dos órgãos competentes a essa área de difícil acesso. A atuação dos profissionais na unidade de saúde dos assentamentos requer uma formação apropriada que instrumentalize a compreensão da realidade vivenciada pela população do campo contemplando a especificidade local. Recomenda-se priorizar a atuação dos profissionais existentes na comunidade, pois estes trazem consigo as experiências e trajetórias vivenciadas e, portanto, são capazes de compreender a dinâmica do trabalho da mulher e homem no/do campo, bem como a distância dessa territorialização para o acesso a esse serviço.

No presente estudo, os trabalhadores em saúde realizaram reflexões pertinentes e criaram perspectivas para novas atitudes inseridas no contexto da saúde, como a vacinação, o cuidado com os usuários, os próprios problemas também vivenciados por serem, em sua maioria, moradores de assentamento. A roda de conversa permitiu também que fossem elencados elementos na promoção da saúde, como novas regras da vacina contra o HPV, que se somaram ao conhecimento já adquirido. Dessa forma, pode-se afirmar que a roda de conversa não pode ser considerada somente uma forma de coleta de dados, mas, sobretudo, um processo educativo com equalização de conhecimentos e tomadas de decisão pelos participantes.

Os resultados encontrados revelam a necessidade de uma análise criteriosa dos fatores que podem ter influenciado para a adesão vacinal abaixo do recomendado com vistas a uma reestruturação da estratégia da política nacional de vacinação para a população alvo. Já foi demonstrado que práticas de intervenção para aumentar o conhecimento dos profissionais de saúde sobre a epidemiologia do HPV e sua relação com o câncer são essenciais para aumentar a taxa de recomendação e adesão à vacina, podendo melhorar o comportamento com outras imunizações^14^.

A ação conjunta entre os setores de educação, saúde e população é necessária para que a população se conscientize sobre a importância da imunização contra o HPV^27 , 30^. A formação dos profissionais deve instigar seu comprometimento com o objetivo de pautar políticas públicas voltadas para a assistência à saúde da população da reforma agrária. A realidade observada neste estudo pode servir de base para o melhor entendimento da realidade de outros assentamentos existentes no Brasil.

Essas políticas precisam esclarecer as dúvidas que podem levar à desconfiança e a fatores de antecipação que causam hesitação ou recusa vacinal. Igualmente importante é que a pesquisa local seja conduzida para entender melhor a hesitação na vacinação contra o HPV e outros determinantes da adoção para informar e moldar políticas nacionais.

Estudos que exploram especificamente as intervenções educacionais para profissionais de saúde ainda são limitados, mas sabe-se que enfermeiros e médicos de família são comumente referidos em estudos qualitativos como os que mais influenciam nas decisões de vacinação contra o HPV. Além disso, observa-se também a influência da família, pais de outras crianças e amigos que não vacinaram ou que recomendaram a vacina^30^.

Os resultados encontrados nessa população reforçam a necessidade de ações que promovam o conhecimento sobre o HPV e a vacinação. O fortalecimento da ESF e a educação permanente e continuada dos profissionais, bem como a confecção e distribuição de material explicativo à comunidade, certamente contribuirão para aumentar a confiança dos pais em relação à vacinação de seus filhos.
